# Contribution of the Rapid LAMP-Based Diagnostic Test (RLDT) to the Evaluation of Enterotoxigenic *Escherichia coli* (ETEC) and *Shigella* in Childhood Diarrhea in the Peri-Urban Area of Ouagadougou, Burkina Faso

**DOI:** 10.3390/microorganisms11112809

**Published:** 2023-11-19

**Authors:** Alimatou Héma, Samuel S. Sermé, Jean Sawadogo, Amidou Diarra, Aissata Barry, Amidou Z. Ouédraogo, Issa Nébié, Alfred B. Tiono, Sophie Houard, Subhra Chakraborty, Alphonse Ouédraogo, Sodiomon B. Sirima

**Affiliations:** 1 Groupe de Recherche Action en Santé (GRAS), Ouagadougou 06 BP 10248, Burkina Faso; s.serme@gras.bf (S.S.S.); j.sawadogo@gras.bf (J.S.); a.diarra@gras.bf (A.D.); a.barry@gras.bf (A.B.); oz.amidou@gras.bf (A.Z.O.); i.ouedraogo@gras.bf (I.N.); a.tiono@gras.bf (A.B.T.); a.ouedraogo@gras.bf (A.O.); s.sirima@gras.bf (S.B.S.); 2 European Vaccine Initiative (EVI), Universitäts Klinikum Heidelberg Vossstrasse 2, Geb. 4040, 69115 Heidelberg, Germany; sophie.houard@euvaccine.eu; 3 Department of International Health, Johns Hopkins Bloomberg School of Public Health, Baltimore, MD 21205, USA; schakr11@jhu.edu

**Keywords:** enterotoxigenic *Escherichia coli*, *Shigella*, children under five, RLDT, Burkina Faso

## Abstract

The estimates of enterotoxigenic *Escherichia coli* (ETEC) and *Shigella* burden in developing countries are limited by the lack of rapid, accessible, and sensitive diagnostics and surveillance tools. We used a “Rapid LAMP based Diagnostic Test (RLDT)” to detect ETEC and *Shigella* in diarrheal and non-diarrheal stool samples from a 12-month longitudinal cohort of children under five years of age in a peri-urban area of Ouagadougou in Burkina Faso (West Africa). To allow comparison with the RLDT-*Shigella* results, conventional culture methods were used to identify *Shigella* strains in the stool samples. As conventional culture alone cannot detect ETEC cases, a subset of *E. coli*-like colonies was tested using conventional PCR to detect ETEC toxins genes. Of the 165 stool samples analyzed for ETEC, 24.9% were positive when using RLDT against 4.2% when using culture followed by PCR. ETEC toxin distribution when using RLDT was STp 17.6% (29/165), LT 11.5% (19/165), and STh 8.5% (14/165). Of the 263 specimens tested for *Shigella*, 44.8% were positive when using RLDT against 23.2% when using culture. The sensitivity and specificity of the RLDT compared to culture (followed by PCR for ETEC) were 93.44% and 69.8% for *Shigella* and 83.7% and 77.9% for ETEC, respectively. This study indicates that both *Shigella* and ETEC are substantially underdiagnosed when using conventional culture and highlights the potential contribution of the new RLDT method to improve enteric disease burden estimation and to guide future efforts to prevent and control bacterial enteric infection and disease.

## 1. Introduction

According to the WHO, diarrhea is responsible for the death of around 1300 children worldwide every day [1]. Enteric bacteria are the second most common cause of diarrhea worldwide. Enterotoxigenic *Escherichia coli* (ETEC) and *Shigella* spp. are the most important pathogensu/ bacteria in terms of frequency and severity of the illnesses caused in young children [2]. *Shigella* alone affects 163 million people per year, and the 165,000 cases that occur in children under five years of age result in 55,000 deaths [3,4]. ETEC is responsible for a further 20,000 deaths per year [5]. *Shigella* is an invasive pathogen responsible for bloody and watery diarrhea. ETEC exerts its pathogenicity via two main enterotoxins and colonization factors (CF) [6]. CFs enable ETEC to colonize the small intestine, allowing the expression of one or both of the main enterotoxins in the vicinity of the intestinal epithelium responsible for secretory diarrhea [7]. These two main enterotoxins are heat-stable enterotoxins (ST) and heat-labile enterotoxins (LT) [8]. Among the wide range of CFs, those most commonly present in diarrheal strains include CFA/I, CS3 and CS6 [5,6].

In addition to acute illness, *Shigella* and ETEC are also responsible for long-term consequences on childhood growth and psychomotor development, even in asymptomatic carriers [7,9,10].

The management of diarrhea caused by *Shigella* and ETEC has become more complicated due to the emergence of high-level resistance to commonly used antibiotics [11,12]. Due to their high pathogenicity and increasing antimicrobial resistance, the WHO has designated *Shigella* and ETEC as potentially lethal bacteria and priorities in terms of vaccine research [13].

In most developing countries in sub-Saharan Africa, including Burkina Faso, a lack of suitable technical laboratory resources, including fieldable diagnostics for surveillance, considerably underestimates the burden of bacterial diarrheas. In these countries, *Shigella* spp. are traditionally diagnosed through the use of culture, followed by identification via biochemical methods. ETEC infections are usually diagnosed by selecting suspected *E. coli* colonies on differential media and using PCR to detect the presence of ETEC-specific genes, such as those encoding LT or ST or by using ELISA [5,14]. Stool culture for *Shigella* is generally only performed in reference hospital laboratories [15]. In these laboratories, ETEC is only tested for research purposes, but not routinely. Assessing the precise burden of diarrhea caused by ETEC and *Shigella* could stimulate and better direct efforts to develop effective vaccines and improve the overall prospects for more effective disease prevention and control. Ideally, diagnostic tests in these endemic countries should be reliable, sensitive, specific, rapid, and accessible. Currently, the Rapid LAMP-based Diagnostic Test (RLDT) meets all these criteria; a simple and rapid diagnostic method developed by the Johns Hopkins University team and described by Chakraborty et al. [16] can detect ETEC and *Shigella* directly from fresh or frozen stool samples [17]. This technique has a specificity and a sensitivity comparable to qPCR [15,16,17]. RLDT provides a platform that can be easily adapted to the diagnosis of other infectious diseases in limited-resource settings. In endemic settings like Burkina Faso, limited data exist on the burden of predominant gut bacteria (*Shigella* and ETEC) as determined via highly effective and sensitive methods like RLDT.

In this study, we aimed to determine the feasibility of implementing RLDT in Burkina Faso and further assess the contribution of *Shigella* and ETEC to the childhood diarrheal disease burden in Africa using a potentially more sensitive diagnostic tool than culture-dependent methods.

## 2. Materials and Methods

### 2.1. Study Design and Participants

The analysis reported here was conducted on a subset of stool samples collected during an observational study entitled “Study on the Burden of Shigellosis in Africa as a Prelude to Clinical Evaluation of an Oral Vaccine Candidate” (NCT04312906). From December 2020 to March 2021, children under five years of age from a peri-urban community in Ouagadougou were enrolled and followed for 12 months to document the occurrence of diarrhea. Follow-up of the last participant included in the study ended in March 2022. Children were included if their families intended to remain in the study area for at least 12 months from enrolment. Children with congenital or severe neonatal disease were excluded. A subset of culture-positive and culture-negative *Shigella* stool samples was selected by convenient sampling (diarrheal and non-diarrheal stools) for RLDT analysis of *Shigella* and ETEC.

### 2.2. Laboratory Methods

- Collection of stool specimens, isolation, and identification of *Shigella* and ETEC

Stool samples were initially processed using conventional culture methods for the detection of bacterial enteropathogens, and an aliquot of the samples was stored at −80 °C. For the diagnosis of *Shigella* and ETEC when using culture, diarrhea and non-diarrhea stool samples were transported in Cary–Blair transport media at 2–8 °C and inoculated onto MacConkey and xylose–lysine–deoxycholate agars. Suspected *Shigella* colonies were confirmed using API20E and BD Phoenix M50.

To compare ETEC detection when using RLDT and culture, a subset of *E. coli*, like lactose-fermenting colonies selected and screened from MacConkey agar, were confirmed to be ETEC-positive using a multiplex colony PCR assay targeting three heat-stable and heat-labile toxin genes (LT, STh and STp).

DNA extraction was performed through the use of a boiling DNA extraction method [18]. Briefly, DNA was extracted from *E. coli* colonies that were 18–24 h old, harvested from MacConkey agar, suspended in 100 μL of sterile water, and then incubated for 10 min at 100 °C. The supernatant was collected and stored in the freezer at −20 °C until use.

ETEC virulence genes (LT, STp and STh) were screened through the use of conventional PCR using validated DNA primers (Table 1).

The multiplex PCR was optimized to a final volume of 20 µL using PCR in 2.5 mM MgCl2 and 1 µM each of forward and reverse primers (Table 1) and 1 µL of the extracted DNA template. The PCR program used for amplification consisted of 2 min at 95 °C initial denaturation, followed by 30 cycles of 15 s at a 95 °C denaturing temperature, 8 s at a 52 °C annealing temperature, and 10 s at a 72 °C extension temperature. Then, it further underwent 2 min at a 72 °C final extension temperature. This protocol was optimized according to the method described by Connor et al. (2022) [15].

- ETEC and Shigella RLDTs analysis.

Description of the RLDT [16]

Sample preparation: A selection of frozen stool samples, diarrheic and non-diarrheic, selected from positive and negative *Shigella* cultures, were tested using RLDT (flow chart below). Briefly, samples were added to a sample processing tube with lysis buffer followed by lysis. After mixing (by finger tapping), the tube was squeezed to release the lyzed sample, followed by incubation in a dry bath at 100 °C for 5 min.

Description of Shigella-RLDT: amplification was performed in lyophilized reaction tubes (LRT). Tubes were packaged in strips of 8 from A to H as presented in Table 2.

These LRTs allow four *Shigella* samples to be tested per run. The LRTs contain a master mix of primers and fluorescent dye as well as all the reagents required for the lyophilized test for *Shigella ipaH* and RLDT assay inhibitor control.

After the addition of the pre-prepared sample and gentle mixing by tapping with a finger, the strips were incubated for 40 min in a real-time fluorometer reader (Agdia Inc., Elkhart, IN, USA). The results were read as positive/negative from the reader.

Description of ETEC-RLDT: the same extract used for *Shigella*-RLDT was used for ETEC-RLDT. The extract was added to the ETEC-RLDT LRTs. Each strip consists of 8 tubes, organized into two reaction tubes each for the LT, STh and STp genes. One reaction tube was added as an RLDT inhibitor control. Readings were taken in the same way as for *Shigella*-RLDT. Contents of each LRT tube for ETEC are presented in Table 3.

### 2.3. Statistical Analysis

Descriptive statistics, such as proportion, mean, and standard deviation, were used to summarize the baseline characteristics.

Analytical methods to compare two diagnostic tests through the following indicators: sensitivity, specificity, positive predictive value, negative predictive value, area under the curve, and Cohen’s kappa.

*p*-values < 0.05 were considered statistically significant for all statistical analyses.

Comparison of the assays: Sample positivity for ETEC and *Shigella* was compared using RLDT and culture (followed by conventional PCR for ETEC) to determine sensitivity and specificity. A sample was considered positive for ETEC when at least one of the LT, STh, or STp genes (ETEC toxin genes) was positive. Sensitivity and specificity values were expressed as percentages.

The 2006 WHO child growth criteria were used to compute z-scores to determine the nutritional status.

All analyses were performed using Stata MP version 17 (StataCorp, College Station, TX, USA).

## 3. Results

### 3.1. Stool Samples Used for Shigella and ETEC Testing

For *Shigella* detection using both RLDT and culture, a total of 263 samples were included in the analysis. The mean age of children from whom the samples were collected was 24.5 months (±14.24). In this sample, 51.5% were male, and 60% were symptomatic, with 4.9% of symptomatic cases presenting with severe disease according to a modified Vesikari severity score. Approximately 48% of the samples were from children over 24 months of age. Stunting was observed in 25.9% of the children whose samples were tested (Table 4).

For ETEC detection using both RLDT and culture followed by conventional PCR, a total of 165 samples were included in the analysis. The mean age was approximately 24 months (24.17 months +/− 13.50). In this sample, 47.9% were male, and 63.0% were symptomatic, with 3.9% of symptomatic cases presenting with severe disease according to a modified Vesikari severity score. Around 47% of samples were from children over 24 months of age. Stunting was observed in 29.1% of the children whose samples were tested (Table 4).

### 3.2. Stool Samples Positivity for Shigella and ETEC According to Diagnostic Tool

In the original study, there were 2290 stool specimens, of which 72 were culture-positive for *Shigella*. Here, we tested 57 culture-positive and 206 culture-negative stool specimens. These 263 stool specimens were tested using RLDT for the *ipaH* gene. While *Shigella* was detected via culture in 23% of the stool specimens (57/263), RLDT detected *Shigella* in 44.9% (118/263; *p* < 0.001). The highest proportion was found in children aged 12 to 59 months using RLDT and in children over 24 months when using culture. The proportion of *Shigella*-positive cases detected via RLDT was higher than those using culture when characteristics such as age, gender, type of stools, nutritional status (stunting) and severity score were considered. The difference was statistically significant (*p* < 0.001) (Table 5).

Regarding ETEC detection, the proportion of positive samples was around 25% with RLDT and around 4% with culture. The highest proportion was observed in children under 12 months of age by both RLDT and culture. The proportion of ETEC-positive cases detected via RLDT was higher than those detected using culture when characteristics such as age, gender, nutritional status (stunting), and severity score were considered. The difference was statistically significant (*p* < 0.05). However, despite the fact that the proportion detected using RLDT was higher compared to culture for non-diarrheal stools (16.4% vs. 3.3%), the difference was not statistically significant (*p* = 0.192) (Table 5).

- Coinfection of *Shigella* and ETEC

Among stool samples tested simultaneously using RLDT for ETEC and *Shigella* (*n* = 137), we noted a *Shigella*–ETEC coinfection rate of 11.0% (*n* = 15). The distribution of ETEC toxins in coinfection with *Shigella* was as follows: LT (*n* = 4), STp (*n* = 3) and STh (*n* = 8).

### 3.3. ETEC Toxins Genes Distribution using RLDT

Among the stool samples tested using RLDT for ETEC, a proportion of 24.9% was found, i.e., 41 cases of ETEC identified among the 165 samples tested. ETEC with heat-stable porcine toxin (STp) genes had a frequency of 29/165 (17.6%), making them the dominant genes. This was followed by heat-labile toxin (LT) genes 19/165 (11.5%) and human heat-stable toxin (STh) genes 14/165 (8.5%).

**Figure 1:**
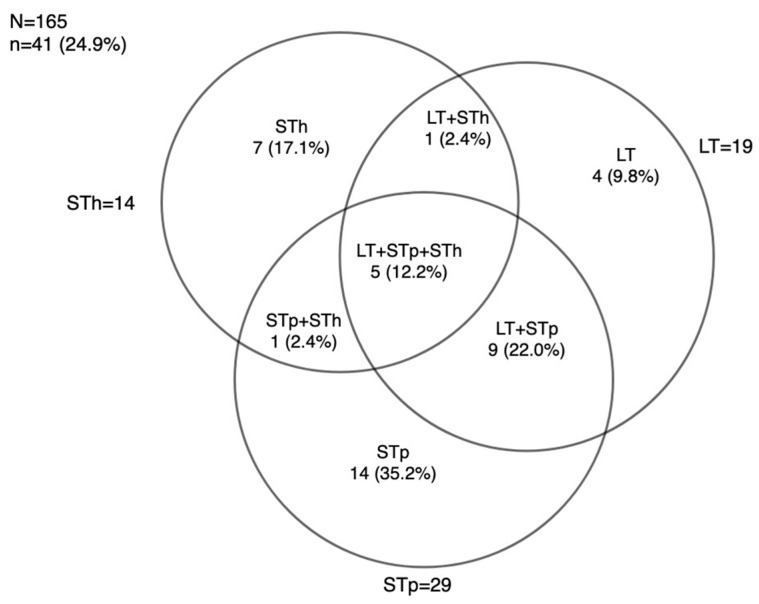
ETEC toxin distribution when using RLDT.

Of the 41 cases of ETEC found, the frequency of ETEC combining three toxins LT + STh + STp was 5/41 (12.2%), as shown in Figure 1. Of the STp cases detected, STp was the only toxin identified in 14/41 cases (34.1%), and it was found in association with LT in nine cases (22.0%) and STh in one case (2.4%). LT was the only toxin identified in four of forty-one cases (9.8%), and LT was associated with STh in only one case. STh was the only toxin identified in seven cases (17.1%). The distribution of ETEC toxins using RLDT is shown in Figure 1.

### 3.4. Performance of the RLDT against Culture for Shigella and ETEC Detection

- *Performance of RLDT against culture for Shigella detection*

The performance of RLDT against culture for *Shigella* detection is shown in Table 6. The evaluation of the sensitivity, specificity, Positive Predictive Value (PPV), and Negative Predictive Value (NPV) of the RLDT using culture as the gold standard is presented in Table 6. The prevalence of *Shigella* was 23.2% when using culture and 44.8% when using RLDT. The sensitivity, specificity, PPV, and NPV of the RLDT with a 95% confidence interval were 93.4% (90.5–96.4%), 69.8% (64.3–75.4%), 48.3% (42.3–54.3%), 97.2% (95.3–99.2%), respectively (Table 6).

**Figure 2:**
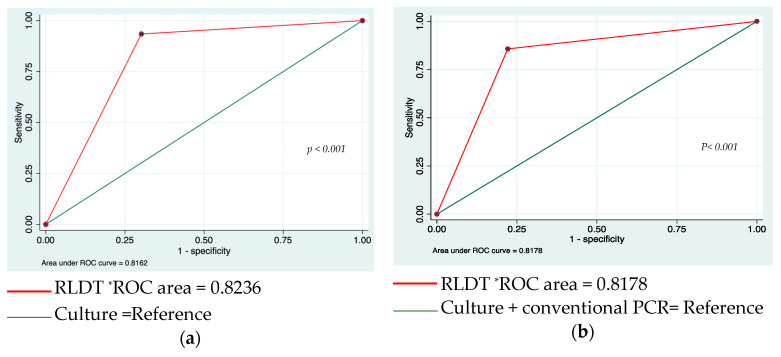
Comparison of RLDT and culture for *Shigella* and ETEC using AUC analysis. (**a**): Comparison of RLDT and culture for *Shigella* detection using AUC analysis. (**b**): comparison of RLDT and culture for ETEC detection using AUC analysis. Note: * Statistical significance (*p* < 0.05). (**a**) ROC curve (receiver operating characteristic curve) for the detection of *Shigella* via RLDT versus culture. (**b**) ROC curve for detection of ETEC using RLDT versus culture. The curves were plotted by calculating the sensitivity and 1-specificity of RLDT compared to culture for *Shigella* (curve a) or ETEC (curve b).

We compared the Area Under the Curve (AUC) for the RLDT and culture in detecting *Shigella* using the culture as a reference. A significant difference between the RLDT and culture performances was observed (*p*-value < 0.001) (Figure 2a).

The analysis of the concordance between stool culture and RLDT in detecting *Shigella* is shown. We obtain a kappa coefficient of 0.48. A kappa coefficient between the RLDT and culture performances was observed (*p*-value< 0.001), and there is a moderate concordance (Table 7)

This section presents the analysis of the concordance between stool culture and RLDT in detecting ETEC. We obtain a kappa coefficient of 0.19. A kappa coefficient between RLDT and culture performance was observed (*p*-value < 0.001). There is a slight concordance (Table 8).

- *Performance of the RLDT against culture followed by PCR for* ETEC *detection*

The performance of the RLDT against culture for ETEC is shown in Table 9. The evaluation of ETEC sensitivity, specificity, PPV, and NPV of the RLDT using culture are presented in Table 9. The prevalence of ETEC was 4.24% when using culture and 24.9% when using RLDT. The sensitivity, specificity, PPV, and NPV of the RLDT with a 95% confidence interval were 83.7% (80.4%, 91.1%), 77.9% (71.5%, 84.2%), 14.6% (9.2%, 20.0%), 99.2% (97.8%, 100.6%), respectively (Table 9).

We also compared the Area Under the Curve (AUC) for the RLDT and culture in detecting ETEC using culture (followed by PCR) as a reference. A significant difference between the RLDT and culture performances was observed (*p*-value < 0.001) (Figure 2b).

## 4. Discussion

This study allowed us to estimate the burden of *Shigella* and ETEC and assess the feasibility of the implementation and performance of RLDT in the context of a country endemic for these enteric pathogens. Fieldable, sensitive, and specific rapid diagnostics for the identification of enteric pathogens in endemic areas like Burkina Faso have long been needed, so this study addresses a critical need in diagnostic technology.

In previous studies carried out in Ouagadougou, Burkina Faso, the prevalence of ETEC was low, ranging from 3.2% to 12.9% [19,20,21,22]. This prevalence is similar to that obtained via culture (followed by conventional PCR) in the present study, where a proportion of 4.2% was found.

These low prevalence rates could be explained by the low sensitivity of culture [4]. In these studies, ETECs were also detected through the use of multiplex PCR on *E. coli* colonies isolated via culture. In contrast, ETEC detection via RLDT shows a higher proportion. Indeed, the ETEC detection rate when using RLDT at 24.9% (*n* = 165) was similar to that of a study in Zambia reporting ETEC prevalence when using RLDT at 22%, which had 91 to 100% sensitivity and specificity when compared to quantitative PCR results.

In this study, we also found that among circulating ETEC strains, STp-ETEC strains were the most prevalent, followed by LT-ETEC and LT + STp-ETEC strains. The least detected ETEC strain was STh-ETEC. A similar distribution of toxin genes among ETEC strains was also reported by Bonkoungou et al. [20] in Ouagadougou, Burkina Faso (ST 7.0%, LT 4.2% and ST + LT 1.7%). We found that within age groups, children aged over 24 months were most at risk of ETEC infection, with a prevalence of around 12.2%. In this study, STp-ETEC strains were found more often (~2 times) in symptomatic cases than in non-diarrheal samples. Similarly, the GEMS and MAL-ED studies showed that higher proportions of ST-producing ETEC were more frequent than LT-producing ETEC in the first and second years of life [5].

In the present study, we also evaluated the performance of RLDT for *Shigella* and ETEC detection compared with conventional culture. Overall, the sensitivity and specificity of RLDT for ETEC (83.3%, 81.4%) and *Shigella* (93.4%, 69.8%) were higher compared to culture. The high sensitivities and specificities of RLDT tested for ETEC and *Shigella* compared with culture in this study confirm the high performance of RLDT [15,16,17]. The lower sensitivity of culture could be due to several factors inherent in the requirements of this technique. In culture-based methods, the viability of pathogens in the sample taken is essential, whereas enteric bacteria such as ETEC and *Shigella* survive poorly in the external environment [15,23,24]. Thus, if bacteria die during transport or storage prior to culturing, this can lead to false-negative results. Although transport and storage media such as buffered glycerin saline or Cary Blair medium are used to increase the survival time of these bacteria, viability is difficult. RLDT, on the other hand, can detect the presence of target DNA from both viable and non-viable pathogens. Additionally, the cultivation of enteric bacteria such as ETEC and *Shigella* is extremely time-consuming due to the proliferation of commensal bacteria. This makes it difficult to isolate and accurately identify the pathogen. RLDT specifically targets the genetic markers of the pathogen, minimizing interference from other organisms [15]. It should also be noted that bacterial isolation via culture may require a higher load, whereas during the early stages of infection or in asymptomatic cases, the pathogen load may be relatively low; therefore, RLDT may be more sensitive in detecting even low levels of target DNA.

This study reaffirms the importance of culture-independent tools for the diagnosis and epidemiological surveillance of ETEC and *Shigella*. It is important to note, however, that although RLDT offers greater sensitivity for the detection of *Shigella* and ETEC, culture-based methods nevertheless remain valuable for the isolation and characterization of bacterial pathogens and the monitoring of antibiotic resistance [5]. Genetic and phenotypic characterization of antimicrobial resistance is only possible through culture. In addition, culture-based methods provide a basis for a more detailed antigenic characterization of circulating strains and, in this way, can also help drive vaccine development.

In endemic and resource-limited areas such as Burkina Faso, the combination of several diagnostic approaches, including accessible and rapid molecular methods such as RLDT, can provide a more complete understanding of infectious diseases and aid patient management. Indeed, in developing countries, culture is only performed in specialized laboratories in tertiary-level hospitals due to insufficient technical resources and the high cost of reagents and equipment and the cold chain required for stool culture. In addition, the enormous workload does not allow the laboratories to increase the chances of isolating *Shigella* by examining many suspect colonies, which would increase the sensitivity of the culture method [15]. Given RLDT’s excellent performance in detecting ETEC and *Shigella*, widespread deployment of RLDT in all medical centers in developing countries for ETEC and *Shigella* screening of diarrhea cases, even in the most peripheral establishments, would enable better care of the poorest populations and reduce antibiotic consumption. RLDT is a simple, easy-to-use tool that could even be used by community health workers. As the RLDT is rapid (<1 h), it can be used as a screening test for the rapid management of diarrhea cases. Given the low cost of reagents, its ease of use and as the cold chain is not required, the RLDT would be the ideal tool for ETEC and *Shigella* surveillance in endemic countries, which is essential for obtaining meaningful data on the burden of disease to inform policy-makers and health professionals for the development of control and prevention programs. RLDT-positive samples from peripheral health center laboratories could be stored in preservation media such as Cary–Blair, and then forwarded to specialized laboratories for the isolation of ETEC and *Shigella* colonies.

There were several limitations to this analysis. (1) RLDT uses the ipaH gene as a marker for *Shigella* detection. This gene target, ipaH, is also found in enteroinvasive *E. coli*, and the methods in this study do not distinguish between these similar pathogens. Nevertheless, it is generally thought that *Shigella* is much more widespread and is, therefore, likely to account for most of the ipaH-associated organisms that have been detected. (2) The detection of colonization factors and whole genome sequencing will be performed in the future using the stored isolates.

## 5. Conclusions

This study provided an update on the burden of ETEC and *Shigella* in children under five in Burkina Faso and highlights the important role that RLDT can play in facilitating the diagnosis and surveillance of these enteropathogens. The high burden of ETEC and *Shigella* in our setting calls for continued efforts to improve diagnostics and surveillance for enteric pathogens by screening all diarrhea cases with more sensitive and rapid tools such as RLDT. From this perspective, we could say that an assessment of the cost-effectiveness of this RLDT is justified to inform public health stakeholders regarding its wider use and adoption.

## Figures and Tables

**Table 1:** Primers used to detect ETEC enterotoxins.

ETEC Toxin/Virulence Factor	Target Gene	Primer Name	Primer Sequences (5′ to 3′)	Amplicon Size (bp)
LT	*eltB*	LTF	ACG GCG TTA CTA TCC TCT C	274
		LTR	TGG TCT CGG TCA GAT ATG TG	
STp	*estA1*	STpF1	TCT TTC CCC TCT TTT AGT CAG	166
		STpR2	ACA GGC AGG ATT ACA ACA AAG	
STh	*estA2*	SThnyF	TTCACCTTTCCCTCAGGATG	120
		SThnyR	CTATTATTCATGCTTTCAGGACCA	

**Table 2:** Contents of each lyophilized reaction tube (LRT) for *Shigella*.

Well	A	B	C	D	E	F	G	H
Gene	ipaH	control	ipaH	control	ipaH	control	ipaH	control

**Table 3:** Contents of each lyophilized reaction tube (LRT) for ETEC.

Well	A	B	C	D	E	F	G	H
Gene	LT	STh	STp	control	LT	STh	STp	control

**Table 4:** Characteristics of children whose stools were used for *Shigella* and ETEC testing.

	*Shigella n = 263 (44.87)*	ETEC *n = 165 (23.19)*
**Characteristics**		
Age (Months)		
** 0–11**	57 (21.7)	38 (23.0)
** 12–23**	81 (30.8)	49 (29.7)
** 24+**	125 (47.5)	78 (47.3)
Gender		
** Male**	134 (51.0)	79 (47.9)
** Female**	129 (49.1)	86 (52.1)
**Type of stool**		
Diarrheal	155 (58.9)	104 (63.0)
Non-diarrheal	108 (41.1)	61 (37.0)
**Severity scoring** (Vesikari score for diarrheal cases)		
Mild	144 (93.0)	98 (24.6)
Moderate	5 (3.2)	2 (24.6)
Severe	6 (3.9)	4 (24.6)
**Stunted (HAZ < −2.0)**		
Yes	67 (25.5)	48 (29.1)
No	196 (74.5)	117 (70.9)

The number in brackets represents the percentage of positive samples.

**Table 5:** Positivity of *Shigella* according to diagnostic tool.

	*RLDT*	*Culture*	
Characteristics	Proportion of Positive *Shigella* using RLDT	Proportion of Positive *Shigella* by Culture	*p*-Value
**Overall**	118 (44.9 *)	61 (23.2 *)	<0.001
**Age (Months)**			
0–11	29 (50.9)	13 (22.8)	<0.001
12–23	41 (50.6)	17 (21.0)	
24+	48 (38.4)	31 (24.8)	
**Gender**			
Male	61 (45.5)	27 (20.2)	<0.001
Female	57 (44.2)	34 (26.4)	
**Type of stool**			
Diarrheal	75 (48.4)	29 (18.7)	<0.001
Non-diarrheal	43 (39.8)	32 (29.6)	
**Severity scoring** (Vesikari score for diarrheal cases)			
Mild	69 (47.9)	28 (19.4)	<0.001
Moderate	2 (40.0)	0	
Severe	4 (66.7)	1 (16.7)	
**Stunted (HAZ < −2.0)**			
Yes	29 (43.3)	13 (19.4)	<0.001
No	89 (45.4)	48 (24.5)	

* The number in brackets represents the percentage of positive samples.

**Table 6:** Sensitivity and specificity of *Shigella* RLDT compared to culture.

*Shigella* Culture (as the Gold Standard) vs. RLDT								
Total Samples Screened	Samples *Positive* via RLDT (%)	Samples *Positive* via Culture (%)	False Positive	False Negative	Sensitivity (95%CI)	Specificity (95%CI)	PPV (95%CI)	NPV (95%CI)
263	118	57	61	4	93.4 (90.5, 96.4)	69.8 (64.3, 75.4)	48.3 (42.3, 54.3)	97.2 (95.3, 99.2)

**Table 7:** Cohen’s kappa RLDT stool culture for *Shigella* detection.

Cohen’s Kappa RLDT Stool Culture for *Shigella* Detection					
*Agreement*	Expected Agreement	Kappa	Std. error	z	*p* Value
75.29%	52.75%	0.4769	0.0548	8.70	<0.001

**Table 8:** Cohen’s kappa RLDT stool culture for ETEC detection.

Cohen’s Kappa RLDT Stool Culture for ETEC Detection					
Agreement	Expected Agreement	Kappa	Std. err.	z	*p* Value
78.18%	73.02%	0.1914	0.0503	3.81	<0.001

**Table 9:** Sensitivity and specificity of ETEC RLDT compared to culture.

ETEC Culture (as the Gold Standard *) vs. RLDT								
Total Samples Screened	Samples *Positive* via ETEC RLDT	Samples *Positive* via Culture	False Positive	False Negative	Sensitivity (95%CI)	Specificity (95%CI)	PPV (95%CI)	NPV (95%CI)
165	41	7	35	1	83.7 (80.37, 91.1)	77.9 (71.5, 84.2)	14.6 (9.2, 20.0)	99.2 (97.8, 100.6)

* Note: culture followed by conventional PCR considered the gold standard.

## Data Availability

The dataset is available from the corresponding author upon reasonable request.

## Abbreviations

EDCTP	European and Developing countries Clinical trial Partnership
ETEC	Enterotoxigenic *Escherichia coli*
LT	Heat-labile enterotoxin
STp	Heat-stable enterotoxin Porcine
STh	Heat-stable enterotoxin Human
PCR	Polymerase chain reaction
RLDT	Rapid LAMP-based diagnostic test
Sd	Standard deviation
WHO	World Health Organisation
